# Notes and News

**Published:** 1895-10-26

**Authors:** 


### Notes and News.

(Collected and Communicated.)

Dr. Hughes Bennett has been elected a consulting physician, and Dr. Leonard Guthrie has been appointed to the charge of in-patients, in addition to that of out-patients, at the Hospital for Epilepsy and Paralysis.

Dr. Duclaux has been appointed to fill the vacant post of director of the Pasteur Institute, with Dr. Roux as sub-director.

The following entries have been made at the metropolitan medical schools and colleges:—

| | 1. | 2. | 3. | Total. | 4.* |
|---|---|---|---|---|---|
| St. Bartholomew's Hospital... | 105 | 62 | 0 | 167 | 20 |
| Charing Cross Hospital ... | 17 | 38 | 28 | 83 | — |
| St. George's Hospital | 50 | 3 | 1 | 54 | 1 |
| Guy's Hospital ... ... | 80 | 32 | 35 | 147 | 37 |
| King's College ... ... | 31 | 71 | — | 102 | 11 |
| London Hospital ... ... | 75 | 63 | — | 138 | 13 |
| St. Mary's Hospital ... ... | 71 | 73 | 1 | 145 | 13 |
| Middlesex Hospital ... ... | 20 | 27 | 9 | 56 | 2 |
| St. Thomas's Hospital ... | 73 | 16 | — | 89 | 2 |
| University College ... ... | 45 | 76 | — | 121 | 82 |
| Westminster Hospital ... | 14 | 8 | 0 | 22 | 4 |

* Column 1 equals full curriculum; 2, special course students; 3, dental students; and 4, the number of students who have joined the various institutions to attend the classes for preliminary scientific instruction, and the numbers given as totals do not, therefore, include these.

Cider, as a beverage, has been looked upon with some suspicion by the richer classes in England. Those who abstain from fear, and not taste, should be reassured by the results of the investigations of two French medical men, MM. Carrion and Cantru, who, amongst other gaseous beverages which they commend for certain peculiarities of digestion, speak most highly of cider. In certain forms of dyspepsia it is said to be very desirable, where the process of digestion is too hurried, and for the gouty it is especially recommended as a corrective of the uric acid diathesis. Gout is held responsible for so large a number of ailments nowadays that cider should be shown much favour, to the advantage of the Worcestershire and Herefordshire apple growers.

The report of the Association for the Oral Instruction of the Deaf and Dumb shows that progress is being made with this most important method of teaching, the only method in fact by which these unfortunate people are enabled to take their place in the world among their fellows. The committee regret that the Education Department are not yet prepared to recognise any school for the deaf as a training college for teachers, and say that it is feared that it will be difficult to induce really good trained teachers to devote a third year for such special training. Since the Blind and Deaf Education Act came into force, a little more than a year ago, her Majesty's inspectors have been actively engaged in visiting the schools and asylums in the country, and by refusing to certify some of them are, in many cases, putting an end to inefficient teaching and forcing school authorities to give deaf children a better education. The deaf are now placed on an equality with those who hear in regard to their claim to be properly educated, but a great deal yet remains to be done to make it possible to give them opportunities for instruction, and, unfortunately, this association, which has done so much to further this end, is above £1,600 in debt. It is to be hoped that friends will rally round the committee in their good work, and that funds will be forthcoming to further this important educational movement, which has for its object the restoration to usefulness and intelligence of a class of people who, without the special form of instruction thus provided for them, would be, and hitherto have been, condemned to practical idiocy. The offices of the association are at 11, Fitzroy Square.

No convalescent home exists in Russia at the present time, but efforts are now being made to establish two such institutions, one in St. Petersburg and one in the country, and to this end a bazaar is to be held at St. Petersburg at Christmas-time. Mr. Woolrych Perowne has published the history of the movement. Two committees, it would appear, one composed of Russian ladies and the other of foreign residents, have been energising in the same direction for some time past, neither being aware of the efforts of the other. Discovering the situation these committees have promptly amalgamated, and combined forces, with the result that the impending bazaar has been decided upon. The foreign embassies are interesting themselves in its success, and each nation will have a stall of its own. It is naturally desired that the English stall shall be all it ought to be, and subscriptions, presents for the stall, or help of any kind is asked towards this end. The Empress Dowager is interesting herself much in this stall, and is herself the president of the committeee. Communications on the subject should be addressed to Mr. Perowne, at 5, Endsleigh Gardens, W.C. It seems astonishing that so necessary a charity as a convalescent home should be altogether lacking in a European country in the year 1895. The contemplated homes are to be open to all, "irrespective of creed or nationality, and are meant for the benefit of the professional classes, such as tutors, governesses, employés in business, of whom very many are English." It is much to be hoped that this movement will meet with a ready response from the Russian people, and that not only in the way of money for the erection of the buildings, but in personal interest, which will have ample opportunities for manifesting itself when the new institutions are accomplished facts.

